# Insomnia Proven to be Associated With Prostate Cancer: A Genetic Correlation Study Incorporating Lifestyle Factors

**DOI:** 10.31083/AP46810

**Published:** 2025-09-23

**Authors:** Xiangyu Chen, Lijun Xie, Xiaoqiang Liu

**Affiliations:** ^1^Department of Urology, Tianjin Medical University General Hospital, 300052 Tianjin, China

**Keywords:** prostate cancer, mental disorder, insomnia, Mendelian randomization analysis

## Abstract

**Background:**

Mental disorders (MDs) are associated with prostate cancer (PCa) outcomes, but the results reported by different studies are inconsistent. Our aim was to explore the causal relationship between 10 MDs and PCa using bidirectional two-sample Mendelian randomization (MR) and multivariable MR (MVMR) analysis.

**Methods:**

Our study was based on summary data from genome-wide association studies (GWAS) of PCa and 10 major MDs in the European population. The genetic locus data used in the analysis included variants associated with PCa and the 10 MDs. Causal estimates were calculated using the inverse-variance weighted (IVW) method, and sensitivity MR techniques, including Cochran’s Q test, MR-Egger regression, and MR pleiotropy residual sum and outlier (MR-PRESSO), were employed to evaluate potential horizontal pleiotropy and heterogeneity. All statistical analyses were conducted using R software.

**Results:**

Our study did not find a causal relationship between PCa and the 10 MDs. In reverse MR analysis, a causal association between insomnia and PCa was found only for insomnia, which reduced PCa risk (odds ratio [OR], 0.9706; 95% confidence interval [CI], 0.9468–0.9951; *p* = 0.0188). However, after MVMR adjustment for habits (cigarette smoking, alcohol intake, coffee intake, and tea intake), this causal relationship no longer existed (OR, 1.011; 95% CI, 0.932–1.096; *p* = 0.795).

**Conclusion:**

This study demonstrated a negative correlation between insomnia and PCa from a genetic perspective. However, such results may be mediated by lifestyle habits and therefore need to be interpreted with caution.

## Main Points

1. Mental disorders are associated with prostate cancer (PCa) but this association is complex. 

2. Mendelian randomization analysis is a novel epidemiological tool for studying the causal relationship between exposure and the clinical outcomes of disease.

3. The results showed a negative correlation between insomnia and PCa.

## 1. Introduction

Prostate cancer (PCa) remains an important public health burden. PCa is the most common cancer diagnosed in US men, accounting for 29% of cancer diagnoses; an additional 288,300 new cases are predicted in 2023 [1]. Methods for the early detection and management of PCa have evolved over the past decade, and randomized controlled trials have demonstrated improved survival rates. Therefore, the early identification of suspected high-risk patients and timely disease intervention while reducing overdiagnosis and overtreatment is particularly important [2].

A mental disorder (MD) is a condition characterized by clinically significant disturbances in an individual’s cognition, emotional regulation, or behavior, reflecting dysfunctions in the psychological, biological, or developmental processes underlying mental functioning. MDs are typically associated with considerable distress or impairment in social, occupational, or other essential areas of life [3]. In 1990, the global estimate for MD cases was 654.8 million (with a 95% uncertainty interval [UI] of 603.6 to 708.1 million), while by 2019, this number had risen to 970.1 million cases (ranging from 900.9 to 1044.4 million). This represents a 48.1% increase in the total number of cases over the 29-year period [4].

The confounding effects of MDs on cancer care have previously been described. Approximately one-third of patients with cancer experience mood disorders during hospitalization. In addition to the burden of living with cancer treatment, receiving a cancer diagnosis is a seriously stressful event [5]. The presence of MDs in patients with cancer can lead to serious problems, including prolonged hospitalization, increased somatic side effects, decreased adherence and engagement, reduced quality of life, and increased mortality rates [6, 7]. While health-related quality of life outcomes following PCa treatment are well documented, there has been less focus on the relationship between PCa and mental health outcomes [8, 9].

PCa patients often experience a wide range of mental health issues beyond anxiety and depression, including insomnia, mood disorders, and cognitive impairment. For example, insomnia is a common complaint among PCa patients, particularly those undergoing treatments such as androgen deprivation therapy (ADT), which can disrupt sleep patterns due to side effects like night sweats and hot flashes. Mood disorders, including bipolar disorder and schizophrenia, have also been reported in PCa patients, though their prevalence and impact on cancer outcomes are less well studied. Additionally, neurological disorders such as Alzheimer’s disease and Parkinson’s disease may co-occur with PCa, potentially due to shared genetic or inflammatory pathways. These mental health issues can significantly impact patients’ quality of life, treatment adherence, and overall survival, underscoring the need for a comprehensive understanding of the relationship between psychiatric/neurological disorders and PCa [10, 11]. The conclusions of studies on this topic vary significantly. For instance, a cross-sectional study of Black patients with PCa found a relatively high prevalence of major depressive symptoms (33%), with an increased likelihood of depression among patients who had undergone radiotherapy (odds ratio [OR] 2.38; 95% confidence interval [CI], 1.02–5.51) [12]. The relationship between psychiatric disorders, neurological disorders, and PCa is complex and influenced by a combination of genetic, environmental, and lifestyle factors. Observational studies have suggested potential associations between these conditions but they are often limited by confounding factors, reverse causality, and biases such as recall bias. For example, patients with PCa may develop psychiatric symptoms such as depression or anxiety due to the stress of diagnosis and treatment, while pre-existing mental health conditions may influence lifestyle behaviors (e.g., smoking and alcohol consumption) that could increase cancer risk. These complexities make it challenging to establish causal relationships using traditional observational methods [13, 14].

Mendelian randomization (MR) is an emerging epidemiological technique that leverages genetic variation as unconfounded instrumental variables (IVs) to investigate the causal links between exposures and disease outcomes [15]. Since genetic variants are randomly assigned at conception and are not influenced by environmental or lifestyle factors, MR minimizes confounding and reverse causality. This method is particularly valuable for studying the bidirectional relationships between psychiatric/neurological disorders and PCa, as it allows us to explore whether these conditions are causally linked or merely correlated due to shared risk factors. By leveraging large-scale genome-wide association study (GWAS) data, MR provides a powerful tool to identify potential causal pathways and inform prevention and treatment strategies [16].

Understanding the causal relationships between psychiatric and neurological disorders and PCa has important implications for both clinical practice and public health. If psychiatric or neurological disorders are found to influence PCa risk, this could lead to the development of targeted interventions to reduce cancer incidence in high-risk populations. Conversely, if PCa or its treatments contribute to the development of mental health conditions, this would underscore the need for integrated care models that address both physical and psychological well-being in cancer patients. By using Mendelian randomization, this study aimed to provide robust evidence of these relationships, which could inform personalized prevention and treatment strategies. The goal of this study was to examine the bidirectional causal relationship between PCa and 10 different MDs through two-sample MR analysis, utilizing published data from GWAS conducted in European populations. The psychiatric and neurological disorders included in this study were selected based on their high prevalence, significant global burden, and well-documented associations with both mental health and cancer outcomes. Specifically, we focused on disorders that have been previously linked to PCa in observational studies or share common biological pathways, such as inflammation, immune dysregulation, or hormonal changes. For example, depression and anxiety are frequently reported in cancer patients and have been hypothesized to influence cancer risk through stress-related mechanisms. Similarly, neurodegenerative disorders such as Alzheimer’s disease and Parkinson’s disease have been associated with systemic inflammation, which may also play a role in cancer development [17, 18]. By including a broad range of disorders, we aimed to comprehensively explore the potential bidirectional relationships between mental health and PCa.

## 2. Methods

### 2.1 Study Design 

**Fig. 1.**
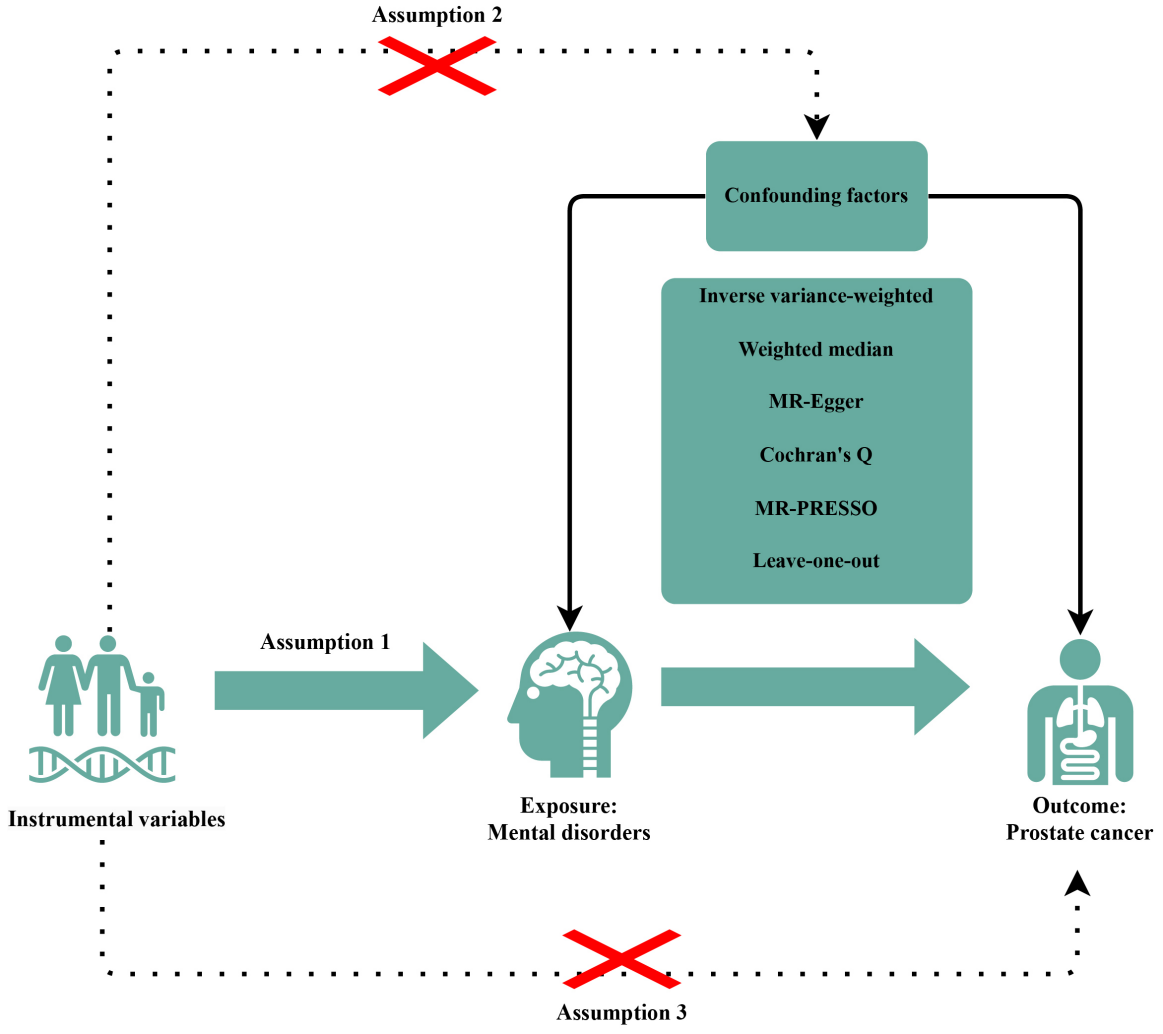
**Diagrams showing the three assumptions of MR analyses and the study design overview**. The “×” indicates that the MR design should follow two assumptions: the IVs are independent of confounding factors between between MDs and PCa; the effects of IVs on the outcomes are solely mediated by the exposures of interest. MR, Mendelian randomization; PRESSO, pleiotropy residual sum and outlier; IVs, instrumental variables; MDs, mental disorders; PCa, prostate cancer.

We systematically assessed the bidirectional causal association between PCa and MDs using a two-sample MR analysis and single nucleotide polymorphisms (SNPs) as IVs. To ensure the accuracy of results, IVs should display these three characteristics: (a) selected IVs must have direct associations with exposure, (b) IVs are independent of confounding factors between MD and PCa, and (c) the effects of IVs on the outcomes are solely mediated by the exposures of interest (Fig. 1).

### 2.2 Data Sources 

We selected PCa as the exposure factor and 10 important psychiatric and neurological disorders as outcome indicators. Simultaneously, we conducted a reverse MR, with 10 psychiatric and neurological disorders as the exposure factors and PCa as the outcome indicator. According to the International Classification of Diseases-10 (ICD-10) classification system [19], we included some important psychiatric and neurological disorders in the study including four neurological disorders: Alzheimer’s disease (G30: Alzheimer disease), epilepsy (G40: Epilepsy), Parkinson’s disease (G20: Parkinson disease), and stroke (G46: Vascular syndromes of brain in cerebrovascular diseases); and six psychiatric disorders: anxiety (F41: Other anxiety disorders), bipolar disorder (F31: Bipolar affective disorder), depression (F32: Depressive episode), insomnia (F51: Nonorganic sleep disorders), mood disorders (F39: Unspecified mood [affective] disorder), and schizophrenia (F20: Schizophrenia).

The genetic data for the MDs phenotype in this study were derived from a comprehensive GWAS meta-analysis focused on European populations. Specifically, data on depression, anxiety, stroke, and epilepsy were obtained from the Medical Research Council-Integrative Epidemiology Unit (MRC-IEU) (https://www.bristol.ac.uk/integrative-epidemiology/), data on bipolar disorder were sourced from the Bipolar Disorder Working Group of the Psychiatric Genomics Consortium (https://pgc.unc.edu/for-researchers/working-groups/bipolar-disorder-working-group/), data on insomnia were sourced from the UK Biobank (https://www.ukbiobank.ac.uk/), data on mood disorders were sourced from the FinnGen consortium R5 release data (https://www.finngen.fi/en/access_results), data on schizophrenia were sourced from the Schizophrenia Working Group of the Psychiatric Genomics Consortium (https://pgc.unc.edu/for-researchers/working-groups/schizophrenia-working-group/), data on Alzheimer’s Disease were sourced from the Alzheimer Disease Genetics Consortium, and (https://www.adgenetics.org/), and data on Parkinson’s disease were sourced from the International Parkinson’s Disease Genomics Consortium (https://gwas.mrcieu.ac.uk/).

Published genetic summary data on PCa were extracted from the largest GWAS meta-analysis, which includes 79,148 cases and 61,106 controls of European ancestry, from the Prostate Cancer Association Group to Investigate Cancer Associated Alterations in the Genome (PRACTICAL) consortium [20]. A comprehensive summary of the data sources can be found in Table 1.

**Table 1.** **Detailed information regarding the data sources of mental disorders and prostate cancer**.

Traits	GWAS ID	Sample size	SNPs	Year	PubMed ID (or URL)	F-statistic
Prostate cancer^a^	NA	140,254	20,346,368	2018	https://www.icr.ac.uk/research-and-discoveries/icr-divisions/genetics-and-epidemiology/oncogenetics/practical	NA
Insomnia^a^	ebi-a-GCST90018869	486,627	24,196,985	2021	34594039	23.5825
Bipolar disorder^a^	ukb-a-83	337,159	10,894,596	2017	https://opengwas.io/	23.3235
Depression^a^	ukb-b-12064	462,933	9,851,867	2018	https://opengwas.io/	23.5710
Anxiety^a^	ukb-b-17243	462,933	9,851,867	2018	https://opengwas.io/	22.6157
Schizophrenia^a^	ieu-b-42	77,096	15,358,497	2014	25056061	22.3041
Mood disorders^a^	finn-b-KRA_PSY_MOOD	218,792	16,380,466	2021	https://opengwas.io/	23.1350
Alzheimer’s disease^a^	ieu-b-2	63,926	10,528,610	2019	30820047	68.2132
Stroke^a^	ukb-b-6358	462,933	9,851,867	2018	https://opengwas.io/	23.0033
Parkinson’s disease^a^	ieu-b-7	482,730	17,891,936	2019	https://opengwas.io/	38.3375
Epilepsy^a^	ukb-b-16309	462,933	9,851,867	2018	https://opengwas.io/	23.5413
Cigarettes per day^b^	ieu-b-25	337,334	11,913,712	2019	30643251	NA
Alcoholic drinks per week^b^	ieu-b-73	335,394	11,887,865	2019	30643251	NA
Coffee intake^b^	ukb-b-5237	428,860	9,851,867	2018	https://opengwas.io/	NA
Tea intake^b^	ukb-b-6066	447,485	9,851,867	2018	https://opengwas.io/	NA

GWAS, genome-wide association studies; SNPs, single nucleotide polymorphisms; NA, not available. ^a^Exposure or outcome in two-sample Mendelian randomization analysis. ^b^Risk factors that may mediate causality between mental disorders and prostate cancer in multivariable Mendelian randomization.

### 2.3 Selection of IVs

The selection criteria for SNPs associated with MDs required a genome-wide significance threshold (*p* < 5 × 10^-6^) and SNPs in linkage disequilibrium (r^2^ < 0.001, with a window size of 10,000 kB) were excluded. Similarly, SNPs related to PCa were selected based on genome-wide significance (*p* < 5 × 10^-8^), with the same linkage disequilibrium exclusion criteria applied. To evaluate the strength of the associations and mitigate potential bias from weak IVs, we computed the F-statistic. A value of F > 10 indicates an absence of weak IV bias, thereby supporting the validity of the association hypothesis. The F-statistic was calculated using the formula: F = beta^2^ / se^2^ [21].

### 2.4 Mendelian Randomization and Statistical Analyses

The inverse variance weighted (IVW) method was employed as the primary analytical approach to estimate the causal relationship between PCa and MDs. The IVW method offers optimal statistical power, provided that all underlying assumptions are satisfied [22]. If more than three SNPs were available, the random effects IVW method was applied; otherwise, the fixed effects IVW method was used. For sensitivity analysis, we employed the weighted median (WM), MR-Egger, and MR pleiotropy residual sum and outlier (MR-PRESSO) methods to address potential bias due to unknown pleiotropy. The weighted median method assumes that the majority of SNPs are valid instruments and is considered robust as long as less than 50% of the IVs exhibit horizontal pleiotropy [23]. The MR-Egger method is considered reliable when more than 50% of the SNPs exhibit horizontal pleiotropy [24]. We employed the MR-PRESSO test to identify pleiotropic SNPs, correct for horizontal pleiotropy by removing outliers, and assess whether there were significant differences in the causal estimates before and after outlier correction using the distortion test [25]. Cochrane’s Q-value was calculated to evaluate the heterogeneity among the selected SNPs. Additionally, individual SNP analysis and leave-one-out analysis were performed to assess the contribution of individual SNPs to the observed associations. To further investigate the causal relationship between insomnia and PCa risk, we adjusted for the effects of lifestyle factors (such as cigarette consumption, alcohol intake, and coffee and tea consumption) using multivariable Mendelian randomization (MVMR). The lifestyle factors selected for this study—cigarette smoking, alcohol consumption, coffee intake, and tea intake—were chosen based on their well-documented associations with both mental health disorders and PCa. Cigarette smoking and alcohol consumption are established risk factors for various cancers, including PCa, and have also been linked to mental health conditions such as depression and anxiety. Coffee and tea intake were included due to their potential protective effects against PCa, as well as their influence on sleep patterns and mental health. These factors were selected to account for potential confounding effects that could mediate the relationship between mental health disorders and PCa. Additionally, we acknowledge that other lifestyle factors, such as physical activity, diet quality, and body mass index (BMI), could also play a role in the relationship between mental health and PCa. However, due to limitations in the availability of genetic IVs for these factors in the datasets used for this study, we were unable to include them in the current analysis. Future studies with access to more comprehensive genetic data should consider incorporating these additional lifestyle factors to provide a more holistic understanding of the potential confounding effects. All MR analyses were conducted using R software (version 4.3.1; R Foundation for Statistical Computing, Vienna, Austria) and its packages “TwoSampleMR (version 0.6.8; https://github.com/MRCIEU/TwoSampleMR)” and “MRPRESSO (version 1.0; https://github.com/rondolab/MR-PRESSO)”. Statistical significance was assumed when *p* < 0.05.

## 3. Results

### 3.1 Positive Causal Association Analysis for PCa and MDs

After excluding abnormal SNPs, SNPs associated with each of the 10 MDs were screened in the PCa database (**Supplementary Table 1**). In our study, all F-statistics were greater than 10, indicating that the instrumental variables were not weak. This suggests that weak instrument bias is unlikely, thereby reinforcing the reliability of our results (Table 1).

In the heterogeneity test, the result of the causal relationship between PCa and mood disorders was *p* < 0.05; therefore, the random effects model in IVW was used for the main analysis. Next, the MR-Egger regression and MR-PRESSO methods were employed to detect horizontal pleiotropy (**Supplementary Table 2**). Although the MR-Egger regression test did not test the horizontal pleiotropy, the MR-PRESSO method showed schizophrenia and mood disorders existed in the horizontal pleiotropy (*p* < 0.001; *p* = 0.01). After removing the outlier SNPs (rs1634741, rs3131785, rs56103503, and rs878987 for schizophrenia), the causal effect of schizophrenia (OR, 0.9815; 95% CI, 0.9529–1.0109; *p* = 0.2166) was still consistent with previous results (**Supplementary Table 1**). However, we did not find significant outliers for mood disorders.

In this study, IVW, WM, and MR-Egger were used to analyze the causal effect. The IVW method results did not reveal any significant causal association between PCa and the 10 MDs. The other two methods further supported the results (**Supplementary Table 1**). Additionally, a “leave-one-out” analysis was conducted on the entire dataset. After sequentially removing each SNP, no single SNP was found to significantly impact the robustness of the results, indicating that the findings are stable.

### 3.2 Reverse Causal Association Analysis for MDs and PCa

After excluding abnormal SNPs, SNPs associated with PCa were screened in the MDs database (**Supplementary Table 3**). In our analysis, all F-statistics exceeded 10, indicating that the instruments employed were robust. This suggests that weak instrument bias is unlikely, thereby supporting the reliability of the results.

In the heterogeneity test, the causal relationship between schizophrenia, mood disorders, Alzheimer’s disease, Parkinson’s disease, and PCa yielded a *p*-value of less than 0.05 (**Supplementary Table 2**). Consequently, the random effects model in the IVW method was applied for the primary analysis. Additionally, the MR-Egger regression and MR-PRESSO techniques were employed to assess potential horizontal pleiotropy (**Supplementary Table 2**). While the MR-Egger regression test did not indicate the presence of horizontal pleiotropy, the MR-PRESSO method revealed significant horizontal pleiotropy for schizophrenia, mood disorders, Alzheimer’s disease, and Parkinson’s disease (*p* < 0.001, *p* = 0.0350, *p* < 0.001, and *p* < 0.001, respectively). After removing the outlier SNPs (rs11210892, rs11658257, rs12062861, rs1233578, rs12691307, rs12829524, rs450735, and rs56873913 for schizophrenia; rs3135293 for mood disorders; rs17125924, rs35695568, and rs3740688 for Alzheimer’s disease; and rs35265698 for Parkinson’s disease), the causal effects of schizophrenia (OR, 1.0029; 95% CI, 0.9800–1.0263; *p* = 0.8025), mood disorders (OR, 0.9566; 95% CI, 0.9022–1.0143; *p* = 0.1373), Alzheimer’s disease (OR, 0.9913; 95% CI, 0.9708–1.0122; *p* = 0.4101), and Parkinson’s disease (OR, 1.0103; 95% CI, 0.9849–1.0363; *p* = 0.4305) on PCa were still consistent with previous results (**Supplementary Table 3**).

**Fig. 2.**
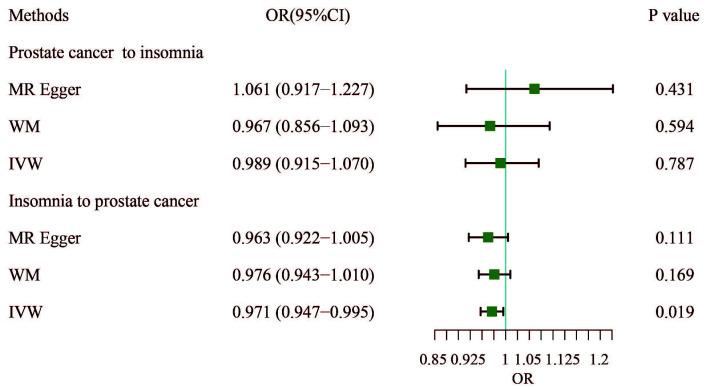
**Results of bidirectional analysis of the association between prostate cancer and insomnia**. WM, weighted median; IVW, inverse-variance weighted; OR, odds ratio; CI, confidence interval.

**Fig. 3.**
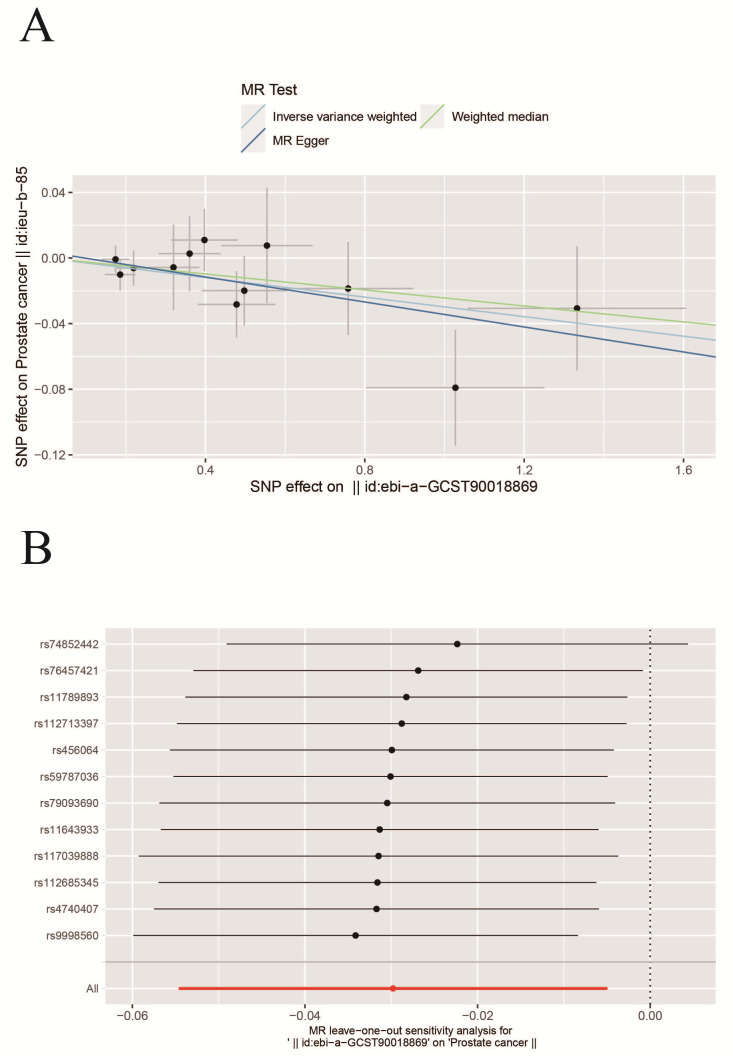
**Scatter and Leave-one-out plots**. (A) Scatter plot of the causal relationship between insomnia and prostate cancer. The slope of each line represents the causal relationship of each method. (B) Leave-one-out plot of the causal relationship between insomnia and prostate cancer.

In this study, IVW, WM, and MR-Egger were used to analyze the causal effect. The IVW method results showed a negative correlation between insomnia and PCa (OR, 0.9706; 95% CI, 0.9468–0.9951; *p* = 0.0188) but the causal effect disappeared regarding the other nine MDs on PCa. The other two methods further supported the results (**Supplementary Table 3**, Fig. 2). Additionally, a “leave-one-out” analysis was conducted on the entire dataset. After sequentially removing each SNP, no single variant was found to significantly impact the robustness of the results, indicating the stability of the study’s findings (Fig. 3).

### 3.3 Multivariable MR Analysis

**Fig. 4.**
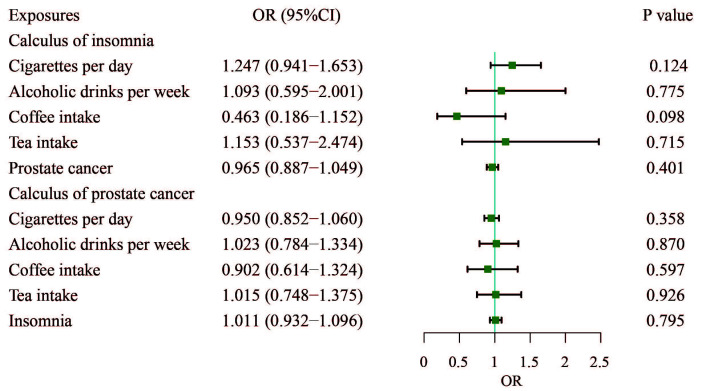
**Adjusted causal effects of cigarettes per day, alcoholic drinks per week, coffee intake, tea intake, and insomnia on the risk of prostate cancer by MVMR analyses**. MVMR, multivariable Mendelian randomization.

To explore whether the protective effect of insomnia on PCa is mediated by factors such as cigarettes smoked per day, alcoholic drinks consumed per week, coffee intake, or tea consumption, we conducted an MVMR analysis to control for these lifestyle variables. The results from the MVMR analysis showed that none of these habits including cigarettes per day, alcoholic drinks per week, coffee intake, or tea consumption were significantly associated with PCa (Fig. 4). Insomnia did not remain the significant protective effect on PCa after adjusting for habits (OR, 1.011; 95% CI, 0.932–1.096; *p* = 0.795).

## 4. Discussion

This study aimed to assess the bidirectional causal relationship between PCa and MDs using two-sample MR. Our findings suggest that insomnia is negatively associated with PCa, indicating that insomnia may reduce the risk of developing PCa. However, we observed no causal relationship between PCa and insomnia. Furthermore, the bidirectional MR analysis did not reveal any causal associations between PCa and conditions such as bipolar disorder, depression, anxiety, schizophrenia, mood disorders, Alzheimer’s disease, stroke, Parkinson’s disease, or epilepsy.

Mental health disorders are a leading cause of disability worldwide and the presence of comorbid health conditions significantly contributes to the overall increase in healthcare costs [26]. A national cohort study has shown that patients with cancer have a sharply increased risk of developing MDs in the years before and after diagnosis [27]. The substantial rise in risk in the year prior to diagnosis may be attributed to the influence of pre-diagnostic cancer symptoms and the intense stress associated with undergoing clinical evaluation for suspected malignancy. Meanwhile, the sharp increase in risk immediately following cancer diagnosis aligns with previous research highlighting the heightened risk of cardiovascular disease and suicide during the period immediately after a cancer diagnosis [27, 28, 29, 30]. Compared with patients with other cancers, patients with PCa have a less elevated risk before and after diagnosis, which may be related to increased knowledge of the disease’s benign prognosis in the general population [31]. Intriguingly, patients with a first diagnosis of an MD have an increased risk of cancer-specific death after developing cancer (hazard ratio (HR), 1.41; 95% CI: 1.06–1.89) [32]. A meta-analysis has also confirmed that depression and anxiety are associated with increased incidence risks, a higher cancer-specific mortality risk, and an increased all-cause mortality risk for PCa [33]. However, most current research on the effects of MDs on PCa has focused only on anxiety and depression.

Our study found a negative association between insomnia and PCa, suggesting that insomnia may be associated with a reduced risk of PCa. However, this finding should be interpreted with caution, as it contradicts previous studies [34, 35] that have linked sleep disturbances to increased cancer risk. One possible explanation for this result is that insomnia may lead to increased healthcare utilization, prompting earlier detection of PCa through routine screening. Alternatively, the observed association may be influenced by unmeasured confounding factors, such as caffeine intake or other lifestyle behaviors, which were not fully accounted for in our analysis. Further research is needed to explore the biological mechanisms underlying this relationship and to determine whether insomnia truly has a protective effect against PCa or if the observed association is due to other factors. For many patients, insomnia onset is triggered by their diagnosis of PCa and they often experience unrelenting and chronic insomnia. Cancer survivors frequently state that sleeplessness is more difficult to manage than their diagnosis and treatment [36]. In studies investigating factors that affect the health-related quality of life of patients with PCa, insomnia consistently ranks high [37, 38]. Patients with PCa are one to two times more likely to experience insomnia symptoms compared with those without PCa [39, 40, 41]. A large prospective study found that patients who slept 3–5 and 6 hours per night respectively have a 64% and 28% higher relative risk of lethal PCa compared with those patients who slept 7 hours per night during the first 8 years of follow-up [42]. Younger patients with PCa are more prone to develop insomnia compared with those without cancer. This may imply that younger men struggle more to accept their new normal [43].

The occurrence of insomnia symptoms in patients with PCa can be a result of cancer treatment. Common treatments for localized PCa include prostatectomy and external radiation, both of which increase the risk of urinary symptoms that may lead to disturbed sleep. According to previous research, 31.5% of patients who underwent prostatectomy experience symptoms of sleeplessness [41]. In a sample of patients undergoing radiation therapy, radical prostatectomy, or brachytherapy, 32% reported symptoms of insomnia, with radiotherapy recipients reporting more severe symptoms [44]. Hormone therapy, particularly ADT, is also associated with higher insomnia scores. ADT for PCa has deleterious effects on sleeping patterns due to the occurrence of night sweats and hot flushes [45]. Sleep problems may also be associated with psychological factors, such as depression or anxiety. Insomnia is no longer viewed solely as a symptom of cancer; it is now widely recognized as an independent risk factor for adverse physical and mental health outcomes [46]. Insomnia is linked to a twofold increased risk of depression, with emerging evidence suggesting that insomnia may precede and contribute to the onset of depressive episodes [47]. In addition, high levels of anxiety may perpetuate sleep difficulties, especially as PCa survivors develop a fear of recurrence [48].

The biological mechanisms underlying the potential link between insomnia and PCa are complex and not yet fully understood. In cancer patients, sleep disturbances are often associated with the activation of inflammatory responses. Tumors produce interleukin-1β, which promotes non-rapid-eye-movement (REM) sleep, and this cytokine also influences various neurotransmitters involved in sleep regulation. Additionally, elevated levels of interleukin-6 (IL-6) and tumor necrosis factor α (TNFα) in PCa patients are linked to an increase in slow-wave sleep. Hormones such as leptin, which are implicated in cancer cell proliferation, can also stimulate the production of IL-6 and TNFα. Furthermore, tumors may induce alterations in serotonergic, dopaminergic, gamma-aminobutyric acid (GABA)-producing, and noradrenergic pathways, all of which contribute to the development of sleep disorders [49, 50].

However, most of the large case-series studies over the past 10 years do not support a correlation between insomnia and the development of PCa [51, 52, 53]. Correspondingly, due to the lack of knowledge of the causal relationship between insomnia and cancer and the lack of routine sleep evaluation by clinicians, insomnia is mainly treated with hypnotic drugs [54]. Our study found that insomnia is negatively associated with PCa, which differs from previous studies. This may be explained by the fact that sleep disturbances or insomnia due to nocturia motivates the patient to seek a comprehensive screening of the urinary system so that an appropriate diagnostic and treatment plan can be developed, which may reduce the incidence of PCa [55, 56, 57, 58]. Age also needs to be taken into account, as the data on insomnia patients used for the analysis were not age-stratified, which may have allowed the lower PCa incidence in young insomnia patients to influence our results [59]. Additionally, we observed that the causal relationship between insomnia and PCa was no longer significant after performing MVMR analysis, which adjusted for the effects of cigarette smoking, alcohol consumption, coffee intake, and tea consumption. Caffeine, the most widely consumed psychoactive substance globally, is commonly found in both coffee and tea, and its potential influence on sleep patterns may confound the relationship between insomnia and PCa [60]. Our study did not find a significant causal relationship between depression, anxiety, and PCa, which contrasts with some previous studies [61, 62] that have reported associations between these mental health disorders and cancer outcomes. This discrepancy may be due to the inherent strengths of MR in minimizing confounding and reverse causality. Unlike an observational study [63], MR relies on genetic variants as instrumental variables, which are less likely to be influenced by environmental or behavioral factors. Additionally, the genetic architecture of depression and anxiety may differ from that of PCa, leading to a lack of detectable causal effects in our analysis. Future studies with larger sample sizes and more comprehensive genetic data may help clarify these relationships. For schizophrenia, bipolar disorder, and mood disorders, our study did not find significant causal associations with PCa. This may be due to the lack of overlapping genetic variants between these psychiatric disorders and PCa, or it may reflect differences in the underlying biological pathways. For example, while schizophrenia has been linked to immune dysregulation and inflammation, these pathways may not directly influence PCa risk. Similarly, for neurological disorders such as Alzheimer’s disease and Parkinson’s disease, the lack of significant findings in our study may reflect the complex and multifactorial nature of these conditions, which may not share common genetic risk factors with PCa. Future research should explore these relationships further, particularly in diverse populations and with larger sample sizes. Caffeine typically increases sleep latency, reduces total sleep time and sleep efficiency, worsens perceived sleep quality, and contributes to the development of insomnia [64]. However, phytochemical compounds (e.g., diterpenes, melanoidins, and polyphenols) contained in coffee and tea play a beneficial role in the prevention of PCa, including the inhibition of oxidative stress and oxidative damage; these actions may play a role, especially in the early stages of transformation of normal cells into malignant tumors [64, 65]. Thus, the reduced risk of PCa in patients with insomnia may be due to the intake of coffee and tea leading to insomnia while producing a chemopreventive effect on PCa. Although the mechanisms connecting insomnia and PCa remain unclear, our study offers a foundation for future exploration of cancer survivors’ experiences with insomnia, its effects on daily functioning, and its management during cancer treatment. Addressing sleep disorders associated with cancer diagnosis and treatment, while educating patients on effective management strategies and improving sleep health, is crucial for advancing cancer care [66].

Depression and anxiety are considered to be worrying comorbidities in the context of PCa. It is estimated that 27% of PCa patients experience anxiety, while 17% are diagnosed with depression [67]. Compared with men without a lifetime history of cancer, men with a history of PCa have twice the odds of screening positive for mild, moderate, or severe symptoms of depression or anxiety [68]. Age is an important sociodemographic factor influencing psychiatric health in PCa patients. Current research indicates that younger adults with cancer are at a higher risk of experiencing psychological distress compared with older adults with cancer. Specifically, younger age is associated with an increased risk of both depression and anxiety [69, 70]. This may be due to different coping styles at different ages: given that younger patients may be more active in their sexual and social lives, they may feel more anxiety if they experience such disease-specific symptoms. For older cancer patients, a decreased emphasis on an externally-focused perspective may help explain the reduction in anxiety and overall distress. This shift could be linked to a greater focus on internal coping mechanisms and acceptance, which may contribute to better emotional adjustment in the face of cancer [69, 71]. Furthermore, ADT has been shown to make patients more susceptible to neurocognitive and psychiatric complications [72, 73]. A study utilizing the TRICARE insurance database found that ADT was significantly associated with an increased incidence of depression (12% vs 7.1%) and dementia (7.4% vs 3.4%) compared with non-ADT regimens (all *p* < 0.001) [74]. Another prospective study demonstrated a significant positive association between ADT duration and Patient Health Questionnaire-9 (PHQ-9) score in depressed patients, indicating that longer ADT duration was associated with more severe depressive symptoms [75]. Cumulatively, the increased risk of MDs associated with ADT in PCa patients may negatively impact their medication adherence and exacerbate their overall condition.

Studies to date on the link between psychiatric and neurological disorders and PCa have been inconsistent. An East Asian cohort study found that Parkinson’s disease was a risk factor for most cancers, including PCa (HR, 1.80; 95% CI, 1.52–2.13; *p* < 0.05) [76]. This finding contrasts sharply with the results observed in most cohort study and meta-analyses conducted in Western populations [77]. This indicates that the results from Western populations cannot be applied to East Asians, and the combined effect of different genetic backgrounds and environmental exposures may be responsible for the different results. The link between Alzheimer’s disease and cancer is also controversial. It has been reported that individuals with Alzheimer’s disease have a 50% reduced risk of developing cancer, while those with cancer, or who have recovered from cancer, exhibit a 35% lower risk of developing Alzheimer’s disease [78]. However, Lin *et al*. [79] found that individuals with PCa were 1.53 times more likely to have a prior diagnosis of Alzheimer’s disease. More experimental evidence is needed in the future to demonstrate the biological pathways and exact mechanisms between psychosis and tumor progression, and to interpret the results of these epidemiological studies.

Several limitations of our study should be acknowledged. First, the metabolite data primarily originated from European populations, which limits the generalizability of our findings across different ethnic groups. Second, while our study addressed a broad range of MDs, the underlying functions and mechanisms of some MDs in relation to disease are not yet fully understood, which may constrain the interpretation of the MR analysis results. Finally, the study did not identify a causal relationship between certain MDs and PCa, highlighting the need for new GWAS to uncover additional MD-related SNPs.

Nonetheless, our study has several strengths. First, unlike previous studies [80, 81] that have typically examined unidirectional causality (MD to PCa or PCa to MD), our study employed a bidirectional MR approach to assess the causal relationship between MDs and PCa. By strictly adhering to the three key assumptions of MR analysis, our study reduced the influence of potential confounders and minimized the risk of reverse causality. Second, we incorporated the most comprehensive set of MD-associated SNPs to date, allowing for a more thorough explanation of the genetic variation underlying MDs. Additionally, because our study utilized large-scale GWAS datasets, the sample sizes for the 10 MDs included were substantially larger than those used in previous studies [13, 34], providing adequate statistical power to assess causality.

## 5. Conclusion

In summary, this study employed the MR method to systematically analyze the causal relationship between PCa and 10 significant psychiatric and neurological disorders from a genetic perspective. The results indicated a negative association between insomnia and PCa, suggesting that insomnia may play a crucial role in the development of PCa. However, due to genetic variations across different ethnicities, countries, and regions, further research is needed to explore these relationships in diverse populations. We anticipate that future GWAS will identify additional genetic variants, enabling MR studies to incorporate more SNPs and larger sample sizes for more robust conclusions. Additionally, future research should consider the impact of lifestyle factors and the age of insomnia patients to better elucidate the underlying mechanisms of the relationship between insomnia and PCa.

## Availability of Data and Materials

The data associated with our study have been deposited into a publicly available repository. The name of the repository and the accession number are IEU OpenGWAS project (https://gwas.mrcieu.ac.uk/); PRACTICAL (https://www.icr.ac.uk/research-and-discoveries/icr-divisions/genetics-and-epidemiology/oncogenetics/practical).

## Supplementary Material

Supplementary material associated with this article can be found, in the online version, at https://doi.org/10.31083/AP46810.
