# Supplementary material for: Insomnia Proven to be Associated With Prostate Cancer: A Genetic Correlation Study Incorporating Lifestyle Factors
Source: Alpha Psychiatry. 2025 Sep 23;26(5):46810. doi: 10.31083/AP46810 (PMC12593758; doi:10.31083/AP46810)
Supplement: Supplementary file 1 [file 2757-8038-26-5-46810-s1.docx]

***Supplementary Material***

**Supplementary Table 1.** Mendelian randomization analyses of prostate cancer on 10 mental disorders.

**Supplementary Table 2.** Using different methods to evaluation the heterogeneity and pleiotropy of prostate cancer and mental disorders.

**Supplementary Table 3.** Mendelian randomization analyses of 10 mental disorders on prostate cancer.

**Supplementary Table 1.** Mendelian randomization analyses of prostate cancer on 10 mental disorders.

| Exposure | Outcome | SNPs | Method | OR (95% CI) | P value |
| --- | --- | --- | --- | --- | --- |
| Prostate cancer | Insomnia | 130 | MR Egger | 1.0605(0.9167–1.2269) | 0.4311 |
|  |  |  | Weighted median | 0.9673(0.8558–1.0933) | 0.5941 |
|  |  |  | Inverse variance weighted | 0.9893(0.9151–1.0695) | 0.7865 |
| Prostate cancer | Bipolar disorder | 127 | MR Egger | 1.0002(0.9996–1.0007) | 0.5153 |
|  |  |  | Weighted median | 0.9999(0.9995–1.0004) | 0.8538 |
|  |  |  | Inverse variance weighted | 0.9999(0.9997–1.0003) | 0.8401 |
| Prostate cancer | Depression | 125 | MR Egger | 1.0012(0.9992–1.0033) | 0.2402 |
|  |  |  | Weighted median | 1.0008(0.9991–1.0025) | 0.3586 |
|  |  |  | Inverse variance weighted | 0.9998(0.9987–1.0009) | 0.7240 |
| Prostate cancer | Anxiety | 114 | MR Egger | 0.9992(0.9980–1.0004) | 0.1715 |
|  |  |  | Weighted median | 0.9997(0.9987–1.0006) | 0.4514 |
|  |  |  | Inverse variance weighted | 0.9997(0.9991–1.0003) | 0.3705 |
| Prostate cancer | Schizophrenia | 130 | MR Egger | 0.9683(0.9062–1.0347) | 0.3435 |
|  |  |  | Weighted median | 0.9685(0.9302–1.0085) | 0.1220 |
|  |  |  | Inverse variance weighted | 0.9868(0.9539–1.0208) | 0.4429 |
|  |  | 126 | IVW after removal of outlier SNPs | 0.9815(0.9529–1.0109) | 0.2166 |
| Prostate cancer | Mood disorders | 129 | MR Egger | 1.0254(0.9764–1.0770) | 0.3172 |
|  |  |  | Weighted median | 1.0285(0.9894–1.0691) | 0.1552 |
|  |  |  | Inverse variance weighted | 0.9868(0.9609–1.0134) | 0.3285 |
| Prostate cancer | Alzheimer's disease | 129 | MR Egger | 0.9995(0.9276–1.0771) | 0.9904 |
|  |  |  | Weighted median | 1.0161(0.9623–1.0730) | 0.5651 |
|  |  |  | Inverse variance weighted | 0.9863(0.9503–1.0236) | 0.4656 |
| Prostate cancer | Stroke | 113 | MR Egger | 0.9999(0.9987–1.0010) | 0.8184 |
|  |  |  | Weighted median | 1.0003(0.9994–1.0012) | 0.5603 |
|  |  |  | Inverse variance weighted | 1.0002(0.9997–1.0008) | 0.4281 |
| Prostate cancer | Parkinson's disease | 126 | MR Egger | 0.9665(0.8911–1.0483) | 0.4126 |
|  |  |  | Weighted median | 0.9857(0.9219–1.0538) | 0.6719 |
|  |  |  | Inverse variance weighted | 0.9964(0.9538–1.0410) | 0.8733 |
| Prostate cancer | Epilepsy | 105 | MR Egger | 0.9996(0.9986–1.0005) | 0.3968 |
|  |  |  | Weighted median | 0.9995(0.9988–1.0002) | 0.1690 |
|  |  |  | Inverse variance weighted | 0.9997(0.9992–1.0001) | 0.1817 |

**Supplementary Table 2.** Using different methods to evaluation the heterogeneity and pleiotropy of prostate cancer and mental disorders.

| Exposue | Outcome | Methods | Q | df | P value for heterogeneity  test | P for MR Egger  intercept | P value for Global test of MR–PRESSO |
| --- | --- | --- | --- | --- | --- | --- | --- |
| Prostate cancer | Insomnia | MR Egger | 105.7079 | 117 | 0.7641 | 0.2710 | 0.7430 |
|  |  | IVW | 106.932 | 118 | 0.7582 |  |  |
| Prostate cancer | Bipolar disorder | MR Egger | 116.6672 | 116 | 0.4651 | 0.3760 | 0.4590 |
|  |  | IVW | 117.4618 | 117 | 0.4706 |  |  |
| Prostate cancer | Depression | MR Egger | 113.7614 | 115 | 0.5151 | 0.1120 | 0.4480 |
|  |  | IVW | 116.3217 | 116 | 0.4741 |  |  |
| Prostate cancer | Anxiety | MR Egger | 114.1355 | 104 | 0.2336 | 0.2860 | 0.2260 |
|  |  | IVW | 115.3997 | 105 | 0.2294 |  |  |
| Prostate cancer | Schizophrenia | MR Egger | 220.4363 | 116 | 1.7445e-08 | 0.5164 | <0.001 |
|  |  | IVW | 221.2411 | 117 | 1.9977e-08 |  |  |
| Prostate cancer | Mood disorders | MR Egger | 150.3822 | 115 | 0.0149 | 0.0710 | 0.0100 |
|  |  | IVW | 154.7112 | 116 | 0.0095 |  |  |
| Prostate cancer | Alzheimer's disease | MR Egger | 110.9622 | 101 | 0.2340 | 0.6870 | 0.2920 |
|  |  | IVW | 111.1421 | 102 | 0.2520 |  |  |
| Prostate cancer | Stroke | MR Egger | 112.6118 | 103 | 0.2432 | 0.4760 | 0.2530 |
|  |  | IVW | 113.1706 | 104 | 0.2533 |  |  |
| Prostate cancer | Parkinson's disease | MR Egger | 137.0146 | 119 | 0.1238 | 0.3840 | 0.1240 |
|  |  | IVW | 137.8948 | 120 | 0.1262 |  |  |
| Prostate cancer | Epilepsy | MR Egger | 72.3472 | 95 | 0.9596 | 0.8140 | 0.9690 |
|  |  | IVW | 72.4029 | 88 | 0.9654 |  |  |
| Insomnia | Prostate cancer | MR Egger | 5.0749 | 10 | 0.8861 | 0.6500 | 0.9040 |
|  |  | IVW | 5.2939 | 11 | 0.9161 |  |  |
| Bipolar disorder | Prostate cancer | MR Egger | 27.0134 | 28 | 0.5175 | 0.3960 | 0.5250 |
|  |  | IVW | 27.7574 | 29 | 0.5309 |  |  |
| Depression | Prostate cancer | MR Egger | 41.0447 | 28 | 0.0532 | 0.6040 | 0.0850 |
|  |  | IVW | 41.4490 | 29 | 0.0629 |  |  |
| Anxiety | Prostate cancer | MR Egger | 3.0485 | 5 | 0.6925 | 0.6220 | 0.7860 |
|  |  | IVW | 3.3243 | 6 | 0.7672 |  |  |
| Schizophrenia | Prostate cancer | MR Egger | 458.7782 | 225 | 3.9519e-18 | 0.4419 | <0.001 |
|  |  | IVW | 459.9878 | 226 | 4.1511e-18 |  |  |
| Mood disorders | Prostate cancer | MR Egger | 48.0959 | 32 | 0.0337 | 0.4150 | 0.0350 |
|  |  | IVW | 49.1227 | 33 | 0.0352 |  |  |
| Alzheimer's disease | Prostate cancer | MR Egger | 79.9526 | 39 | 0.0001 | 0.3190 | <0.001 |
|  |  | IVW | 82.0450 | 40 | 0.0001 |  |  |
| Stroke | Prostate cancer | MR Egger | 1.1637 | 2 | 0.5589 | 0.7550 | 0.7470 |
|  |  | IVW | 1.2914 | 3 | 0.7312 |  |  |
| Parkinson's disease | Prostate cancer | MR Egger | 108.4003 | 48 | 1.4498e-06 | 0.3020 | <0.001 |
|  |  | IVW | 110.8548 | 49 | 1.0862e-06 |  |  |
| Epilepsy | Prostate cancer | MR Egger | 2.5735 | 6 | 0.8602 | 0.3600 | 0.823 |
|  |  | IVW | 3.5575 | 7 | 0.8291 |  |  |

.

**Supplementary Table 3.** Mendelian randomization analyses of 10 mental disorders on prostate cancer.

| Exposure | Outcome | SNPs | Method | OR (95% CI) | P value |
| --- | --- | --- | --- | --- | --- |
| Insomnia | Prostate cancer | 13 | MR Egger | 0.9626(0.9224–1.0047) | 0.1112 |
|  |  |  | Weighted median | 0.9759(0.9427–1.0104) | 0.1688 |
|  |  |  | Inverse variance weighted | **0.9706(0.9468–0.9951)** | **0.0188** |
| Bipolar disorder | Prostate cancer | 30 | MR Egger | 0.1180(3.5279e-05– 394.5747) | 0.6098 |
|  |  |  | Weighted median | 0.2950(2.7227e-04–319.5957) | 0.7320 |
|  |  |  | Inverse variance weighted | 2.0377(1.5279e-02–271.7551) | 0.7755 |
| Depression | Prostate cancer | 31 | MR Egger | 0.7699(0.0063–94.6133) | 0.9159 |
|  |  |  | Weighted median | 3.7478(0.5951–23.6032) | 0.1594 |
|  |  |  | Inverse variance weighted | 2.6145(0.5786–11.8135) | 0.2117 |
| Anxiety | Prostate cancer | 7 | MR Egger | 1363.9509(9.5945e-07–1.9390e+12) | 0.5318 |
|  |  |  | Weighted median | 3.7135(3.4928e-03–3.9481e+03) | 0.7121 |
|  |  |  | Inverse variance weighted | 5.7645(2.8925e-02–1.1488e+03) | 0.5167 |
| Schizophrenia | Prostate cancer | 232 | MR Egger | 1.0378(0.9443–1.1406) | 0.4414 |
|  |  |  | Weighted median | 1.0118(0.9842–1.0400) | 0.4042 |
|  |  |  | Inverse variance weighted | 1.0013(0.9765–1.0268) | 0.9141 |
|  |  | 224 | IVW after removal of outlier SNPs | 1.0029(0.9800–1.0263) | 0.8025 |
| Mood disorders | Prostate cancer | 34 | MR Egger | 1.0552(0.8943–1.2450) | 0.5288 |
|  |  |  | Weighted median | 0.9429(0.8666–1.0259) | 0.1720 |
|  |  |  | Inverse variance weighted | 0.9905(0.9244–1.0612) | 0.7853 |
|  |  | 33 | IVW after removal of outlier SNPs | 0.9566(0.9022–1.0143) | 0.1373 |
| Alzheimer's disease | Prostate cancer | 47 | MR Egger | 0.9887(0.9476–1.0317) | 0.6048 |
|  |  |  | Weighted median | 0.9971(0.9677–1.0274) | 0.8475 |
|  |  |  | Inverse variance weighted | 1.0045(0.9754–1.0345) | 0.7637 |
|  |  | 44 | IVW after removal of outlier SNPs | 0.9913(0.9708–1.0122) | 0.4101 |
| Stroke | Prostate cancer | 4 | MR Egger | 0.0004(1.2863e-17–9.8099e+09) | 0.6649 |
|  |  |  | Weighted median | 0.0114(3.2316e-06–4.0415e+01) | 0.2834 |
|  |  |  | Inverse variance weighted | 0.0865(7.7457e-05–9.6632e+01) | 0.4943 |
| Parkinson's disease | Prostate cancer | 51 | MR Egger | 0.9657(0.9025–1.0334) | 0.3181 |
|  |  |  | Weighted median | 0.9963(0.9633–1.0305) | 0.8316 |
|  |  |  | Inverse variance weighted | 0.9973(0.9673–1.0282) | 0.8625 |
|  |  | 50 | IVW after removal of outlier SNPs | 1.0103(0.9849–1.0363) | 0.4305 |
| Epilepsy | Prostate cancer | 8 | MR Egger | 7.1320e+05(3.5452e-10–1.4348e+21) | 0.4818 |
|  |  |  | Weighted median | 1.2577e–02(4.7072e-06–3.3603e+01) | 0.2770 |
|  |  |  | Inverse variance weighted | 1.7306e–02(2.7804e-05–1.0772e+01) | 0.2165 |
